# Astrocyte-derived clusterin suppresses amyloid formation in vivo

**DOI:** 10.1186/s13024-020-00416-1

**Published:** 2020-11-27

**Authors:** Aleksandra M. Wojtas, Jonathon P. Sens, Silvia S. Kang, Kelsey E. Baker, Taylor J. Berry, Aishe Kurti, Lillian Daughrity, Karen R. Jansen-West, Dennis W. Dickson, Leonard Petrucelli, Guojun Bu, Chia-Chen Liu, John D. Fryer

**Affiliations:** 1 Department of Neuroscience, Mayo Clinic, Collaborative Research Building CR03-010, 13400 E. Shea Blvd, Scottsdale, AZ 85259 USA; 2 Neuroscience Graduate Program, Mayo Clinic Graduate School of Biomedical Sciences, Scottsdale, AZ 85259 USA; 3 Department of Neuroscience, Mayo Clinic, Birdsall Research Building BI235, 4500 San Pablo Rd, Jacksonville, FL 32224 USA

**Keywords:** Alzheimer’s disease, Clusterin, Aβ, Amyloid plaques, Adeno-associated viral vectors, Haploinsufficiency

## Abstract

**Background:**

Accumulation of amyloid-β (Aβ) peptide in the brain is a pathological hallmark of Alzheimer’s disease (AD). The clusterin (*CLU*) gene confers a risk for AD and CLU is highly upregulated in AD patients, with the common non-coding, protective CLU variants associated with increased expression. Although there is strong evidence implicating CLU in amyloid metabolism, the exact mechanism underlying the CLU involvement in AD is not fully understood or whether physiologic alterations of CLU levels in the brain would be protective.

**Results:**

We used a gene delivery approach to overexpress CLU in astrocytes, the major source of CLU expression in the brain. We found that CLU overexpression resulted in a significant reduction of total and fibrillar amyloid in both cortex and hippocampus in the APP/PS1 mouse model of AD amyloidosis. CLU overexpression also ameliorated amyloid-associated neurotoxicity and gliosis. To complement these overexpression studies, we also analyzed the effects of haploinsufficiency of *Clu* using heterozygous (*Clu*^*+/−*^) mice and control littermates in the APP/PS1 model. CLU reduction led to a substantial increase in the amyloid plaque load in both cortex and hippocampus in APP/PS1; *Clu*^*+/−*^ mice compared to wild-type (APP/PS1; *Clu*^*+/+*^) littermate controls, with a concomitant increase in neuritic dystrophy and gliosis.

**Conclusions:**

Thus, both physiologic ~ 30% overexpression or ~ 50% reduction in CLU have substantial impacts on amyloid load and associated pathologies. Our results demonstrate that CLU plays a major role in Aβ accumulation in the brain and suggest that efforts aimed at CLU upregulation via pharmacological or gene delivery approaches offer a promising therapeutic strategy to regulate amyloid pathology.

**Supplementary Information:**

**Supplementary information** accompanies this paper at 10.1186/s13024-020-00416-1.

## Background

Alzheimer’s disease (AD) is the most common form of age-related dementia, currently affecting more than 5 million individuals nationwide [1] . Given the large scale of AD and steady increase in the aging population, providing a better understanding of the pathogenesis of this disease is imperative. Deposition of amyloid-β (Aβ) peptide in the brain is a key initiating event leading to the development of AD [2]. Toxic amyloid aggregates accumulate in the brain in the form of extracellular plaques [3] and are commonly found in leptomeningeal and cortical blood vessels as cerebral amyloid angiopathy (CAA) [4, 5].

Clusterin (CLU), also known as apolipoprotein J (apoJ), is a ubiquitous glycoprotein widely expressed throughout the human body, with very high expression levels observed in the nervous system [6]. As a prominent extracellular chaperone, CLU is involved in heterogenous biological processes, including lipid homeostasis, complement inhibition, cell cycle, and apoptosis [7]. Large-scale genome-wide association studies (GWAS) have identified a significant association of common polymorphisms within the *CLU* gene with risk of developing AD [8, 9]. Initial studies have reported the ability of CLU to form complexes with Aβ [10] and influence its solubility [11–13], thus preventing amyloid fibril formation. In vitro reports have further shown the protective role of CLU against amyloid-mediated neurotoxicity [14]. Subsequently, we and others have demonstrated that complete CLU deletion using global knockout (*Clu*^*−/−*^) mice in different models of amyloidosis has a profound effect on amyloid aggregation and clearance, resulting in reduced total and fibrillar plaques [15–19]. However, as with all studies utilizing complete-null alleles, unknown developmental or compensatory factors could cloud our interpretation of these data with *Clu*^*−/−*^ mice.

Recent studies have suggested that CLU upregulation may play a protective function. Increased CLU levels have been reported in AD-vulnerable brain regions [20] and in the cerebrospinal fluid (CSF) of AD patients [21]. In addition, the association of elevated levels of plasma CLU with severity of AD has also been shown [21]. Importantly, the protective *T* allele of the major *CLU* variant (rs11136000) has been associated with increased CLU levels [8, 22], suggesting a beneficial effect of CLU upregulation. However, other studies have reported conflicting data showing a significant correlation of increased CLU with brain atrophy and rapid clinical progression of AD patients [23]. Thus, it remains unclear whether elevated CLU levels represent a neuroprotective function in AD.

In the present study, we used different in vivo approaches to show that both ~ 30% CLU overexpression in the brain or a ~ 50% reduction have prominent effects on amyloid pathology and gliosis. Specifically, sustained increase of CLU expression in astrocytes via viral delivery in the APP/PS1 mouse model of amyloidosis led to amelioration of amyloid accumulation and Aβ-mediated neurotoxicity and gliosis. We further demonstrated that CLU haploinsufficiency exacerbates amyloid deposition and gliosis in APP/PS1 mice. These findings may have important implications for optimization of amyloid-related treatments in AD, especially strategies aimed at altering the levels of CLU protein.

## Methods

### Animals

APP/PS1 mice aged 8 months and bearing a double mutation in APP and PS1 (APPswe/PS1ΔE9) were used in this study [24]. Littermate breeding strategy of *Clu*^*+/−*^ mice bred to APP/PS1; *Clu*^*+/−*^ mice was also used. Mice were housed in a temperature and humidity-controlled environment under a 12-h light/dark cycle and with free access to food and water. All studies were performed in accordance with *National Institute of Health Guide for the Care and Use of Laboratory Animals* (National Research Council (2011) Guide for the Care and Use of Laboratory Animals (National Academies Press, Washington, DC), 8th Ed.) under the approved protocol from the Mayo Clinic Institutional Animal Case and Use Committee. All analyses included mice of both sexes in accordance with National Institute of Health directives.

### AAV-GFP and AAV-CLU viral production

Viral vector construction and AAV production was performed, as previously described [25]. Briefly, CLU or GFP expression plasmids were cloned into an AAV vector. The constructs were sequence-verified using ABI3730 with Big Dye chemistry (Applied Biosystems, Foster City, CA). AAV vectors expressing GFP and CLU under the control of the glial fibrillary acidic protein (GFAP) promoter to drive expression in astrocytes were co-transfected with AAV2/8 helper plasmids into HEK293T cells. Cells were harvested and lysed in the presence of 0.5% sodium deoxycholate and 50 U/ml Benzonase (Sigma, St. Louis, MO) by freeze-thawing 48 h post-transfection, and the virus was isolated using a discontinuous iodixanol gradient. Quantitative PCR was used to measure the genomic titer of each virus.

### Intracerebroventricular injections

AAV-GFP or AAV-CLU viruses were injected bilaterally into cerebral lateral ventricles of APP/PS1 and WT pups at postnatal day 2 with 2.75E+ 10 viral particles/ventricle. Briefly, postnatal day 2 mice were cryoanesthesized on ice for 5 min via a cold metal plate. The skull was pierced with the 30-gauge needle just posterior to Bregma and 2 μl of AAV virus was injected into the lateral ventricles. Following the injections, pups were placed on the warm pad until they regained normal color and resumed movement.

### Histological analysis

To examine CLU association with amyloid in humans, a brain specimen was obtained from Mayo Clinic Brain Bank from an individual with pathologically confirmed AD (Braak stage 6, Thal stage 5). Paraffin-embedded sections were first cut at 10 μm, then deparaffinized, rehydrated, and subjected to antigen-retrieval in dH_2_O for 15 min under high temperature. Brain sections were incubated overnight with goat anti-CLU antibody (1:50, Santa Cruz) diluted in 0.5% dry-milk in PBS, washed three times in PBS-X and PBS, followed by overnight incubation with secondary antibody (1:250, Jackson ImmunoResearch). Sections were double labeled with the thioflavine-S stain to detect fibrillar amyloid and mounted using Vectashield (Vector Laboratories, Inc.). The images were captured using Zeiss LSM 700 laser scanning confocal microscope.

For histopathological analyses of amyloid accumulation in mouse brain, 8-month-old APP/PS1^AAV-GFP^ and APP/PS1^AAV-CLU^ mice, and APP/PS1; *Clu*^*+/+*^ and APP/PS1; *Clu*^*+/−*^ mice were used. Mice were deeply anesthetized with pentobarbital (100 mg/kg i.p.) and transcardially perfused with phosphate buffered saline (PBS) to expunge blood from cerebrovasculature. After brain removal, cortex and hippocampus from one hemibrain were isolated, frozen on dry ice, and stored at − 80 °C until further processing.

The remaining hemibrain was drop-fixed in 10% neutral buffered formalin (Fisher Scientific, Waltham, MA) overnight at 4 °C followed by switching the brain to 30% sucrose overnight. The hemibrain was cut into 50 μm coronal sections using a freezing-sliding microtome. Brain sections were then washed in PBS, permeabilized for 30 min in PBS-X (with 0.3% Triton-X100) and blocked for 1 h in 1% non-fat dry-milk in PBS-X. Sections were incubated overnight with primary antibodies diluted in 0.5% non-fat dry-milk in PBS. Anti-CLU (1:50; Santa Cruz), anti-GFAP (1:250; Cell Signaling), anti-Iba1 (1:250; Wako), anti-NeuN (1:250; Millipore), anti-Lamp1 (1:100; Developmental Studies Hybridoma Bank), and anti-Aβ (1:1000; MOAB2, Abcam) were used in the study. After overnight incubation, slides were washed with PBS-X and PBS, followed by incubation with secondary antibodies for 2 h at RT. Sections were double labeled with X-34 stain (a Congo red derivative) to detect fibrillar amyloid and mounted using Vectashield (Vector Laboratories). The images were captured using Zeiss LSM 700 laser scanning confocal microscope and Zeiss AxioImager.Z1/ApoTome microscope.

### Presparation of brain lysates

Brain lysates were prepared using a 3-step sequential extraction, as previously described [26]. Briefly, cortex and hippocampus were homogenized in 500 μL and 300 μL, respectively, of Tris-buffered saline (TBS) containing protease and phosphatase inhibitor cocktail. The brain regions were briefly sonicated followed by the ultracentrifugation at 100,000×g for 1 h at 4 °C. The supernatant was transferred to a new Eppendorf tube and stored in − 80 °C as TBS fraction (soluble fraction). The cortical and hippocampal pellets were resuspended in 500 μL and 300 μL, respectively, of TBS with 1% Triton X-100 (TBS-X) with protease and phosphatase inhibitor cocktail, briefly sonicated, and incubated for 30 min at 4 °C with gentle rotation. The incubation was followed by ultracentrifugation at 100,000×g for 1 h at 4 °C. The supernatant was transferred to a new Eppendorf tube and stored in − 80 °C as TBS-X fraction. The final cortical and hippocampal pellets were resuspended in 300 μL and 100 μL, respectively, of 70% formic acid (FA), followed by rotation for 2 h at 4 °C and ultracentrifugation at 100,000×g for 1 h at 4 °C. The final supernatant was neutralized by the addition of 20 volumes of 1 M Tris base and stored in − 80 °C as FA fraction (insoluble fraction).

### Quantitative analysis of CLU and Aβ levels by ELISA

CLU protein levels in the cortex and hippocampus were assessed by specific enzyme-linked immunosorbent assay (ELISA). Plates were coated using the capture antibody (Mouse Clusterin DuoSet ELISA, R&D Systems) and CLU was detected by using the detection antibody (Mouse Clusterin DuoSet ELISA, R&D Systems), followed by the incubation with Super Slow ELISA TMB reagent (Sigma). Total protein concentrations used for normalization were assessed by Bicinchoninic Acid (BCA) Protein Assay kit (Thermo Scientific), according to the manufacturer’s instructions with a standard curve using BSA.

The levels of soluble and insoluble Aβ_40_ and Aβ_42_ were assessed by sensitive sandwich Aβ_40_ or Aβ_42_-specific ELISAs. Plates were coated using human anti–Aβx-40 (13.1.1) and Aβx-42 (2.1.3) capture antibodies. Aβ standards were prepared by using human synthetic Aβ_40_ and Aβ_42_. To detect Aβ species, HRP- conjugated Ab5 secondary antibody was used followed by the incubation with Super Slow ELISA TMB reagent (Sigma) to develop. Protein concentrations were assessed by Bicinchoninic Acid (BCA) Protein Assay kit (Thermo Scientific), according to the manufacturer’s instructions with a standard curve using BSA.

### Quantitative analysis of amyloid deposition and neuritic dystrophy

For stereological analyses 8-month-old APP/PS1^AAV-GFP^ and APP/PS1^AAV-CLU^ mice, and APP/PS1; *Clu*^*+/+*^ and APP/PS1; *Clu*^*+/−*^ were used. As previously described [17], for each animal, 3 coronal sections, separated by 300 μm, were used for the quantification. The cortical and hippocampal regions were marked and StereoInvestigator software (MBF Bioscience) was used to count the percentage of area covered by total and fibrillar amyloid, CAA presence in leptomeningeal and penetrating vessels, and neuritic dystrophy in the cortex and hippocampus.

### Real-time quantitative PCR

Total RNA was isolated using RNeasy Mini Kit (Qiagen) according to the manufacturer’s instructions. Random-primed reverse transcription was performed using High-Capacity cDNA Reverse Transcription kit (Thermofisher) according to manufacturer’s protocol. cDNA was added to a reaction mix (10 μL final volume) containing 300 nM gene-specific primers and Universal SYBR green supermix (Bio-Rad). All samples were run in triplicate and were analyzed on a Quant Studio 7 Flex Real Time PCR instrument (Applied Biosystems; Life Technologies). Relative gene expression was normalized to GAPDH controls and assessed using the 2^-ΔΔCT^ method. Primer sequences are as follows (5′ to 3′): *Gapdh F*: CTGCACCACCAACTGCTTAG, *Gapdh R*: ACAGTCTTCTGGGTGGCAGT, *Gfap F:* GGAGAGGGACAACTTTGCAC, *Gfap R*: AGCCTCAGGTTGGTTTCATC, *Cst7 F:* GCCCTCTGCTGCCTAACTTC*, Cst7 R:* ATCCTGGCTTCACACTGGAG.

### Statistical analyses

Statistical significance of experiments involving two groups was assessed by Student’s *t* test. The statistical analyses of four groups were performed by using two-way ANOVA with the Tukey’s post hoc test. Data are presented as mean ± S.E.M. For all statistical analyses Graphpad Prism 5.04 (Graphpad) software was used.

## Results

### AAV-mediated CLU expression in astrocytes

**Fig. 1:**
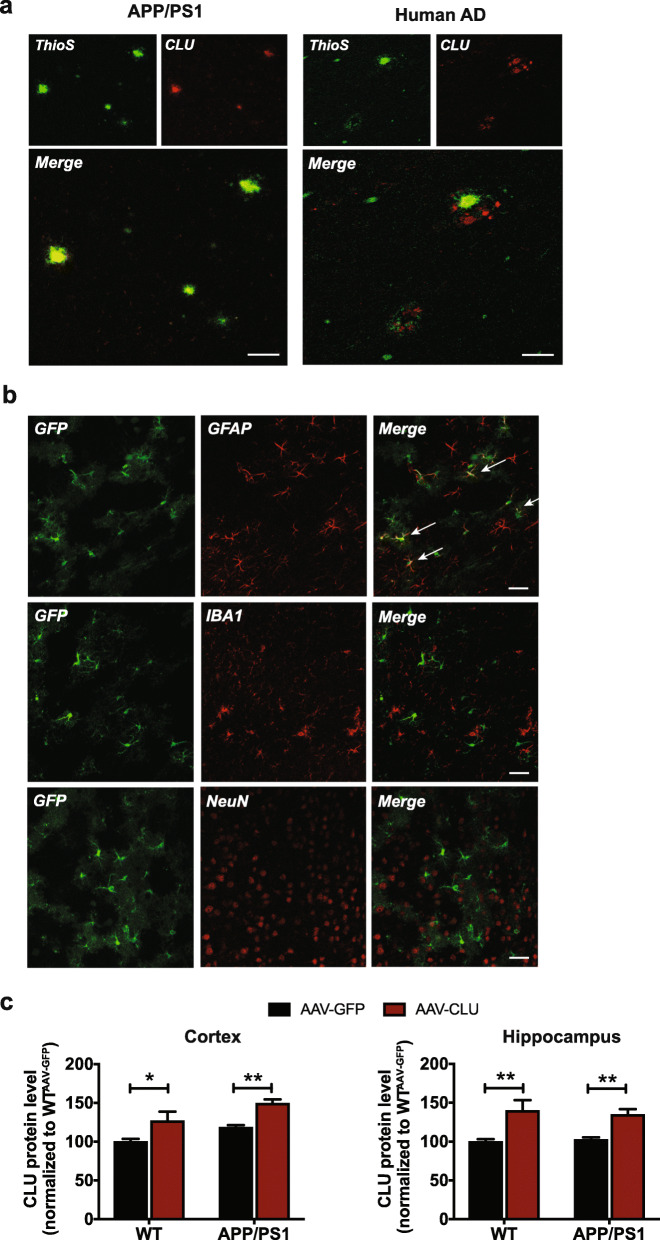
CLU co-localizes with amyloid plaques in mouse models and human AD and is specifically overexpressed in astrocytes. **a** Extensive CLU immunoreactivity (red) was observed in amyloid deposits (green) in cortex of APP/PS1 animals and brain tissue of an AD individual. Scale bar, 50 μm. **b** AAV-mediated specific expression of GFP (green) in GFAP-positive astrocytes (red) but not IBA1-positive microglia (red) or NeuN-specific neurons (red) in APP/PS1 animals. Scale bar, 100 μm. **c** CLU protein levels were assessed in cortex and hippocampus of WT and APP/PS1 mice by enzyme-linked immunosorbent assay (ELISA). Data represent mean ± S.E.M. Cortex: WT^AAV-GFP^: *N* = 6 mice/group (100 ± 3.70), WT^AAV-CLU^: *N* = 6 mice/group (127 ± 11.99), APP/PS1^AAV-GFP^: *N* = 10 mice/group (118 ± 2.98), APP/PS1^AAV-CLU^: *N* = 10 mice/group (150 ± 4.84). Hippocampus: WT^AAV-GFP^: *N* = 6 mice/group (100 ± 3.09), WT^AAV-CLU^: *N* = 6 mice/group (140 ± 13.37), APP/PS1^AAV-GFP^: *N* = 10 mice/group (103 ± 2.96), APP/PS1^AAV-CLU^: *N* = 10 mice/group (135 ± 6.97). Two-way ANOVA with Tukey’s multiple comparisons tests were used, *p < 0.05, **p < 0.01

To investigate the effect of differential CLU levels on amyloid pathology, we first evaluated the CLU localization in APP/PS1 mice and human AD brain. Consistent with previous studies [17], we observed an abundant immunoreactivity of CLU surrounding amyloid plaques in APP/PS1 mice (Fig. 1a) and human AD brain sections (Fig. 1a). We next delivered intracerebroventricular injections of adeno-associated viral (AAV) vectors expressing murine CLU (AAV-CLU) or green fluorescent protein (GFP, AAV-GFP) as control into APP/PS1 and wild-type (WT) littermates at postnatal day 2. Given that astrocytes are the major source of CLU in the brain, we specifically targeted CLU expression in astrocytes by using the glial fibrillary acidic protein (GFAP) promoter. Three months post viral injection we assessed the pattern of GFP expression in the brain. Immunofluorescent analysis showed a widespread GFP immunoreactivity throughout the brains of APP/PS1 (APP/PS1^AAV-GFP^) animals with high levels of GFP expression observed in cortical and hippocampal regions (Fig. S1). In addition, we found an extensive co-localization of GFP with GFAP-positive astrocytes (Fig. 1b) but no co-expression of GFP within microglia (Fig. 1b) or neurons (Fig. 1b), confirming the specificity of viral transduction as has been reported previously with this paradigm [27].

We next evaluated CLU protein levels to determine the degree of overexpression we achieved in cortex and hippocampus of 8-month-old WT and APP/PS1 animals, measured by a CLU-specific enzyme-linked immunosorbent assay (ELISA). Viral injection of AAV-CLU led to a sustained but physiologic CLU overexpression in cortex of WT (*p < 0.05) and APP/PS1 (**p < 0.01) mice of ~ 30% compared to animals injected with control AAV-GFP vectors (Fig. 1c). Similarly, a significant ~ 35–40% increase in CLU levels was detected in hippocampus of WT^AAV-CLU^ (**p < 0.01) and APP/PS1^AAV-CLU^ (**p < 0.01) mice relative to animals injected with control AAV-GFP virus (Fig. 1c). These data show that viral delivery of AAV-CLU leads to a specific yet physiologic overexpression of CLU in astrocytes that persists throughout life.

### CLU overexpression ameliorates amyloid pathology in APP/PS1 mice

**Fig. 2:**
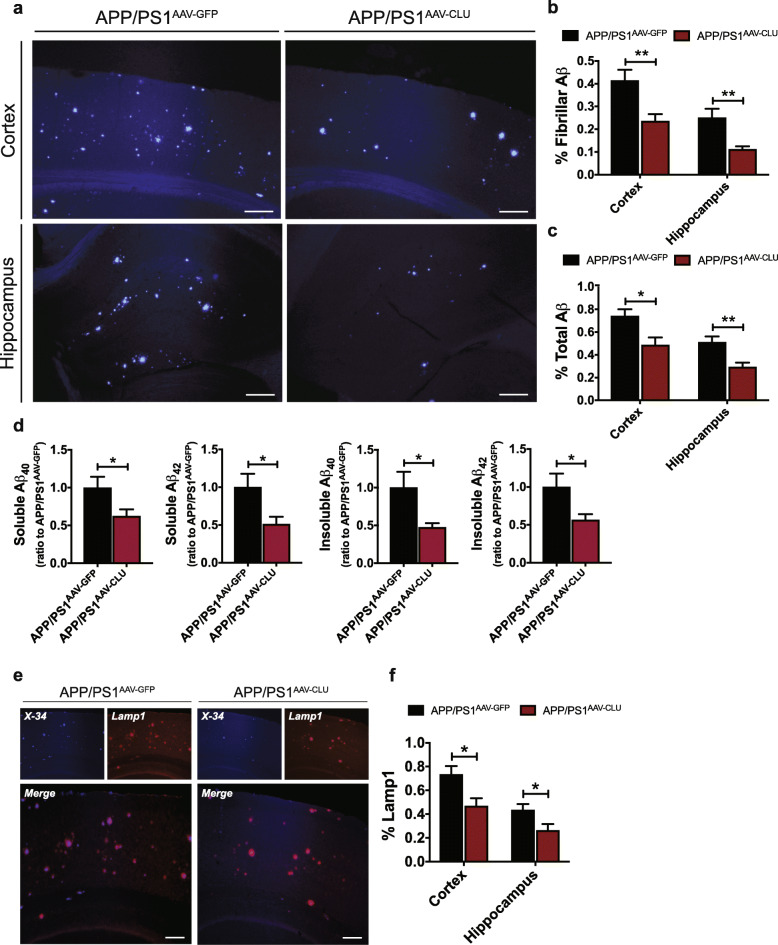
CLU upregulation reduces parenchymal amyloid accumulation in APP/PS1 mice. **a** Representative images of fibrillar amyloid deposition in cortex and hippocampus of 8-month-old APP/PS1^AAV-GFP^ and APP/PS1^AAV-CLU^ animals. Scale bar, 100 μm. **b**-**c** Stereological quantification of (**b**) fibrillar amyloid deposits (Cortex: APP/PS1^AAV-GFP^ (0.41 ± 0.05), APP/PS1^AAV-CLU^ (0.24 ± 0.03); Hippocampus: APP/PS1^AAV-GFP^ (0.25 ± 0.04), APP/PS1^AAV-CLU^ (0.11 ± 0.01)) and **c** total amyloid accumulation (Cortex: APP/PS1^AAV-GFP^ (0.74 ± 0.06), APP/PS1^AAV-CLU^ (0.49 ± 0.07); Hippocampus: APP/PS1^AAV-GFP^ (0.51 ± 0.05), APP/PS1^AAV-CLU^ (0.29 ± 0.04)), *N* = 11–12 mice/group. For each animal three brain sections were analyzed. Data are presented as mean ± S.E.M. and each brain region was analyzed by Student’s *t*, *p < 0.05, **p < 0.01. **d** Quantification of the Aβ_40_ and Aβ_42_ levels in the soluble and insoluble cortical fractions of 8-month-old APP/PS1^AAV-GFP^ and APP/PS1^AAV-CLU^ mice by ELISA. *N* = 10–12 mice/group. Data are presented as mean ± S.E.M.: (soluble Aβ_40_: APP/PS1^AAV-GFP^ (1 ± 0.14), APP/PS1^AAV-CLU^ (0.62 ± 0.09); soluble Aβ_42_: APP/PS1^AAV-GFP^ (1 ± 0.18), APP/PS1^AAV-CLU^ (0.51 ± 0.10)) and insoluble (Aβ_40_: APP/PS1^AAV-GFP^ (1 ± 0.21), APP/PS1^AAV-CLU^ (0.47 ± 0.06); Aβ_42_: APP/PS1^AAV-GFP^ (1 ± 0.18), APP/PS1^AAV-CLU^ (0.56 ± 0.08)). Data analyzed by Student’s *t*, *p < 0.05. **e** Amyloid plaques (blue) were surrounded by dystrophic neurites (red) in APP/PS1^AAV-GFP^ and APP/PS1^AAV-CLU^ mice. CLU overexpression was associated with overall reduction of neuritic dystrophy in APP/PS1 mice. Scale bar, 100 μm. **f** Stereological analysis of Lamp1 labeling in cortex and hippocampus of APP/PS1 mice (Cortex: APP/PS1^AAV-GFP^ (0.73 ± 0.07), APP/PS1^AAV-CLU^ (0.47 ± 0.06); Hippocampus: APP/PS1^AAV-GFP^ (0.43 ± 0.05), APP/PS1^AAV-CLU^ (0.26 ± 0.05)). *N* = 10 mice/group. Data are presented as mean ± S.E.M. and Student’s *t* tests were used to analyze cortex and hippocampus, *p < 0.05

The effect of elevated CLU levels on amyloid deposition in the brain was assessed 8 months after viral injections, when moderate amyloid pathology is observed in this APP/PS1 amyloid model. We performed a neuropathological analysis of cortical and hippocampal regions in APP/PS1^AAV-GFP^ and APP/PS1^AAV-CLU^ mice using, the Congo red derivative, X-34 to label fibrillar amyloid deposits. Importantly, we found that ~ 30% overexpression of CLU led to a significant reduction in the amount of fibrillar plaques in cortex (**p < 0.01) (Fig. 2a, b) and hippocampus (**p < 0.01) (Fig. 2a, b) of APP/PS1^AAV-CLU^ mice compared to controls. Given that amyloid deposits in the brain in the form of both fibrillar and diffuse plaques, we also examined total Aβ accumulation. As with fibrillar amyloid plaques, overexpression of CLU resulted in a decrease of total amyloid deposition in cortex (*p < 0.05) (Fig. 2c, Fig. S2) and hippocampus (**p < 0.01) (Fig. 2c, Fig. S2). However, CLU overexpression in astrocytes did not influence amyloid accumulation in cerebral blood vessels (Fig. S3).

We next extracted the soluble and insoluble Aβ species and determined the levels of Aβ_40_ and Aβ_42_ in cortex of APP/PS1^AAV-GFP^ and APP/PS1^AAV-CLU^ mice by Aβ-specific ELISAs. In agreement with histological data, we found that increased CLU expression substantially influenced the levels of Aβ, with significant reductions of soluble and insoluble pools of Aβ_40_ (*p < 0.05) and Aβ_42_ (*p < 0.05) in APP/PS1^AAV-CLU^ animals compared to controls (Fig. 2d). Together, these findings suggest that sustained CLU overexpression in the brain ameliorates amyloid burden in brain parenchyma of APP/PS1 animals.

### Increased CLU levels influence neurotoxicity and gliosis

Neuritic dystrophy, in the form of severely swollen dendrites and axons, is commonly observed around fibrillar plaques [28]. To determine whether increasing CLU levels influenced the formation of neuritic dystrophy, we performed histological examination with lysosomal-associated membrane protein 1 (Lamp1) labeling to mark dystrophic neurites in proximity to X-34 labeled fibrillar amyloid aggregates. Although Lamp1 immunoreactivity was observed around amyloid plaques in APP/PS1^AAV-GFP^ and APP/PS1^AAV-CLU^ animals, CLU overexpression significantly reduced the overall amount of dystrophic neurites in cortex and hippocampus of APP/PS1^AAV-CLU^ mice relative to their controls (Fig. 2e, f and Fig. S4a). No obvious differences were seen in the number of dystrophic neurites around individual plaques between either group of mice (Fig. S4b).

**Fig. 3:**
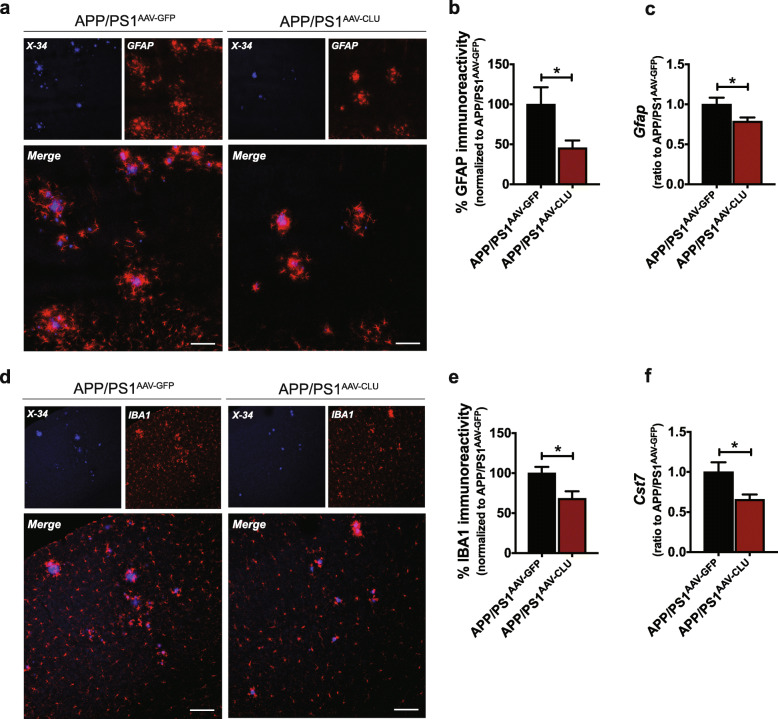
Elevated CLU levels influence amyloid-associated gliosis in APP/PS1 mice. **a** Astrogliosis (red), determined by GFAP staining, was present in close proximity to amyloid plaques (blue) in APP/PS1 mice. Scale bar, 100 μm. **b** Quantification of GFAP immunoreactivity showing the significant decrease in the number of reactive astrocytes in APP/PS1^AAV-CLU^ mice (45.44 ± 9.40) compared to controls (100 ± 21.17). *N* = 10 mice/group. Data are presented as mean ± S.E.M. and analyzed by Student’s *t* test *p < 0.05. **c** Real-time quantitative PCR showing a decrease in the levels of *Gfap* in cortex of APP/PS1^AAV-CLU^ (0.78 ± 0.05) compared to APP/PS1^AAV-GFP^ mice (1 ± 0.08). *N* = 15 mice/group. Data are presented as mean ± S.E.M. and analyzed by Student’s *t* test *p < 0.05. **d** Increased CLU expression decreases the level of microgliosis (red) associated with amyloid plaques (blue). Scale bar, 100 μm. **e** Quantification of IBA1 immunoreactivity presenting a reduction of microgliosis in APP/PS1^AAV-CLU^ (68.32 ± 8.83) compared to APP/PS1^AAV-GFP^ (100 ± 7.91). *N* = 10 mice/group. Data are presented as mean ± S.E.M. and analyzed by Student’s *t* test *p < 0.05. **f** RT-qPCR showing a reduction in *Cst7* levels in cortex of APP/PS1^AAV-CLU^ mice (0.65 ± 0.06) compared to APP/PS1^AAV-GFP^ animals (1 ± 0.12). *N* = 15 mice/group. Data are presented as mean ± S.E.M. and analyzed by Student’s *t* test *p < 0.05

Since abundant gliosis is associated with the presence of amyloid pathology [17], we next assessed whether CLU overexpression had a differential effect on inflammatory changes in APP/PS1 mice. Although reactive astrocytes and microglia, labeled with GFAP and IBA1 immunostaining, respectively, were present in close proximity to amyloid plaques in APP/PS1^AAV-GFP^ and APP/PS1^AAV-CLU^ mice (Fig. 3a, d), we found significant differences in the level of gliosis associated with CLU overexpression. Specifically, GFAP immunoreactivity was reduced in cortex of APP/PS1^AAV-CLU^ mice compared with APP/PS1^AAV-GFP^ mice (Fig. 3a, b and Fig. S4c). Similarly, we found a markedly reduced expression of IBA1 in mice injected with AAV-CLU compared to controls (Fig. 3d, e Fig. S4e). However, the levels of gliosis were not different between groups when normalized to fibrillar amyloid (Fig. S4d, f). Finally, we tested whether CLU upregulation in astrocytes affects gliosis at the molecular level. We observed a significant reduction in the expression of *Gfap* and *Cst7* (encoding cystatin F) transcripts further confirming the CLU effect on astrogliosis and microgliosis, respectively (Fig. 3c, f). Collectively, these results indicate that CLU overexpression is associated with reduction of both neuritic dystrophy and gliosis, which is accompanied by decrease in Aβ accumulation in APP/PS1^AAV-CLU^ mice compared with APP/PS1^AAV-GFP^ mice.

### CLU haploinsufficiency leads to augmented amyloid pathology

**Fig. 4:**
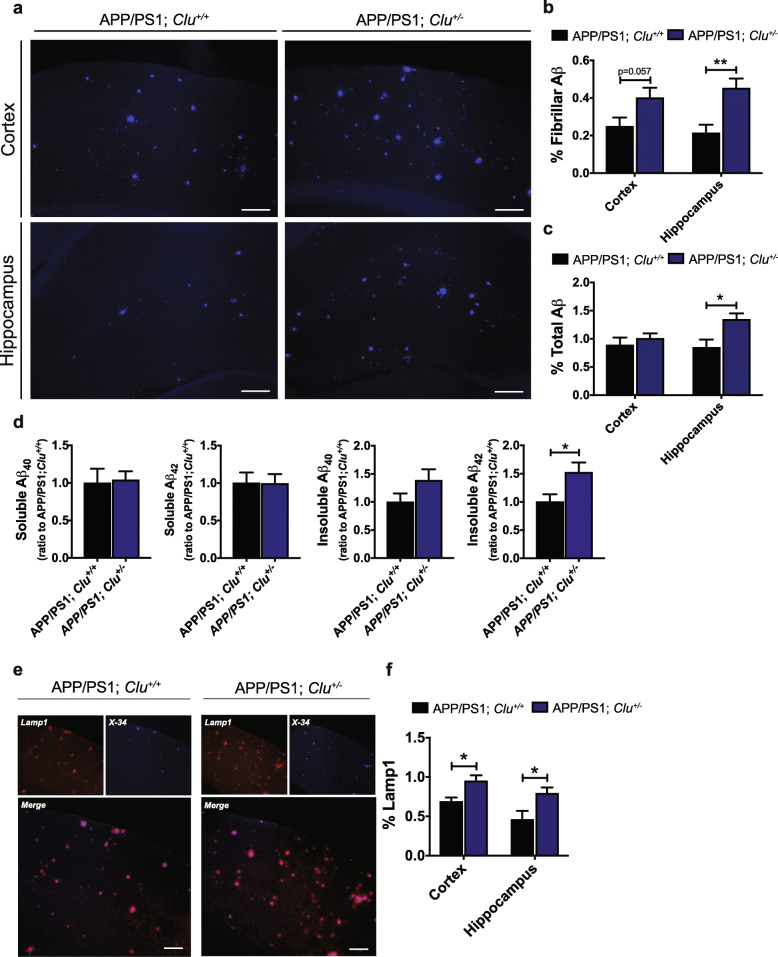
CLU haploinsufficiency leads to exaggerated deposition of amyloid plaques in APP/PS1 mice. **a** Representative images of fibrillar amyloid accumulation in 8-month-old APP/PS1; *Clu*^*+/+*^ and APP/PS1; *Clu*^*+/−*^ mice. Scale bar, 100 μm. **b**-**c** The amount of **b** fibrillar (Cortex: APP/PS1; *Clu*^*+/+*^ (0.25 ± 0.05) and APP/PS1; *Clu*^*+/−*^ (0.40 ± 0.05); Hippocampus: APP/PS1; *Clu*^*+/+*^ (0.21 ± 0.04) and APP/PS1; *Clu*^*+/−*^ (0.45 ± 0.05)) and **c** diffuse amyloid plaques (Cortex: APP/PS1; *Clu*^*+/+*^ (0.89 ± 0.13) and APP/PS1; *Clu*^*+/−*^ (1.01 ± 0.09); Hippocampus: APP/PS1; *Clu*^*+/+*^ (0.85 ± 0.14) and APP/PS1; *Clu*^*+/−*^ (1.34 ± 0.11)) was analyzed in cortex and hippocampus. *N* = 7–8 mice/group. For each animal three brain sections were analyzed. Data are presented as mean ± S.E.M. and Student’s *t* tests were used for each brain region *p < 0.05, **p < 0.01. **d** The levels of soluble and insoluble Aβ in the cortex of APP/PS1 mice. *N* = 7–8 mice/group. Data are presented as mean ± S.E.M.: (soluble Aβ_40_: APP/PS1; *Clu*^*+/+*^ (1 ± 0.19), APP/PS1; *Clu*^*+/−*^ (1.039 ± 0.12); soluble Aβ_42_: APP/PS1; *Clu*^*+/+*^ (1 ± 0.14), APP/PS1; *Clu*^*+/−*^ (0.99 ± 0.13)) and insoluble (Aβ_40_: APP/PS1; *Clu*^*+/+*^ (1 ± 0.15), APP/PS1; *Clu*^*+/−*^ (1.38 ± 0.20); Aβ_42_: APP/PS1; *Clu*^*+/+*^ (1 ± 0.14), APP/PS1; *Clu*^*+/−*^ (1.52 ± 0.18)) Data analyzed by Student’s *t* test *p < 0.05. **e** Higher amount of neuritic dystrophy (red) was observed around amyloid plaques (blue) in APP/PS1; *Clu*^*+/+*^ mice. Scale bar, 100 μm. **f** Stereological analysis of Lamp1 labeling in cortex and hippocampus of APP/PS1; *Clu*^*+/+*^ ((0.69 ± 0.05), (0.46 ± 0.11) and APP/PS1; *Clu*^*+/−*^ mice (0.95 ± 0.07) (0.79 ± 0.07). *N* = 7–8 mice/group. Data are presented as mean ± S.E.M. and each brain region was analyzed by Student’s *t* test *p < 0.05

Given that ~ 30% CLU overexpression significantly influenced amyloid deposition in brain parenchyma, we next wondered whether reducing CLU levels leads to more severe phenotype. To test this, thorough neuropathological examination was performed on 8-month-old haploinsufficient *Clu*^*+/−*^ mice (CLU heterozygotes) crossed to APP/PS1 animals. Notably, APP/PS1; *Clu*^*+/−*^ mice exhibited higher amount of fibrillar amyloid deposited in cortex (*p* = 0.057) and hippocampus (**p < 0.01) compared to APP/PS1; *Clu*^*+/+*^ littermate controls (Fig. 4a, b). Moreover, we noted significantly higher abundance of total Aβ in hippocampus (*p < 0.05) but not cortex of APP/PS1; *Clu*^*+/−*^ mice compared to APP/PS1; *Clu*^*+/+*^ animals (Fig. 4c, S5). Despite a significant increase in amyloid load in brain parenchyma of APP/PS1; *Clu*^*+/−*^ mice, CLU haploinsufficiency did not affect amyloid deposition in cerebrovasculature (Fig. S6). Biochemical examination of the major Aβ forms in cortical tissue showed a significantly higher level of Aβ_42_ in the insoluble fraction of APP/PS1; *Clu*^*+/−*^ mice (*p < 0.05) compared to controls, with no differences observed in the levels of soluble Aβ_40_ and Aβ_42_ (Fig. 4d).

Analysis of Lamp1 immunoreactivity revealed more dystrophic neurites surrounding amyloid plaques in brain parenchyma in APP/PS1; *Clu*^*+/−*^ mice compared to APP/PS1; *Clu*^*+/+*^ animals (Fig. 4e, f and Fig. S7a). No differences between groups were observed in the levels of Lamp1 when normalized to fibrillar amyloid (Fig. S7b).

**Fig. 5:**
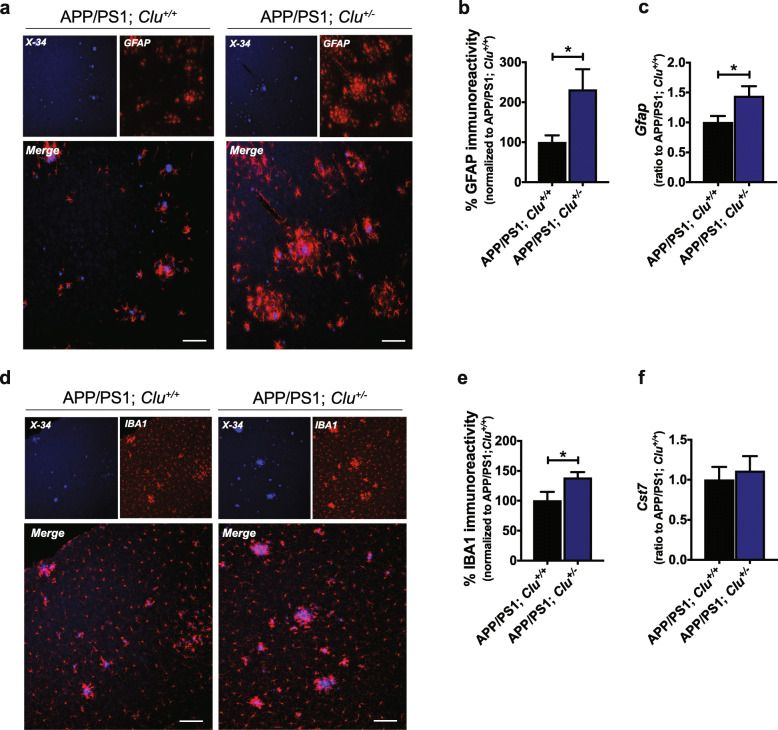
CLU haploinsufficiency results in augmented gliosis. **a** Astrogliosis (red) is more abundant around amyloid aggregates (blue) in APP/PS1; *Clu*^*+/−*^ mice compared to controls. Scale bar, 100 μm. **b** Quantification of GFAP intensity in brain sections of APP/PS1; *Clu*^*+/+*^ (100 ± 17.25) compared to APP/PS1; *Clu*^*+/−*^ mice (231.1 ± 51.71). *N* = 7–8 mice/group. Data are presented as mean ± S.E.M. and analyzed by Student’s *t* test *p < 0.05. **c** Relative expression of *Gfap* levels in cortex of APP/PS1; *Clu*^*+/+*^ (1 ± 0.11) and APP/PS1; *Clu*^*+/−*^ mice (1.44 ± 0.17). *N* = 8 mice/group. Data are presented as mean ± S.E.M. and analyzed by Student’s *t* test *p < 0.05. **d** Microgliosis (red) marked by IBA1 is associated with amyloid plaques (blue). Scale bar, 100 μm. **e** Quantification of IBA1 intensity in brain sections of APP/PS1 mice showing higher levels of microgliosis in APP/PS1; *Clu*^*+/−*^ mice (138.2 ± 9.83) compared to APP/PS1; *Clu*^*+/+*^ mice (100 ± 14.81). *N* = 7–8 mice/group. Data are presented as mean ± S.E.M. and analyzed by Student’s *t* test *p < 0.05. **f** Expression levels of *Cst7* in cortex of APP/PS1; *Clu*^*+/+*^ (1 ± 0.16) and APP/PS1; *Clu*^*+/−*^ (1.11 ± 0.19) mice. *N* = 8 mice/group. Data are presented as mean ± S.E.M. and analyzed by Student’s *t* test

Additionally, APP/PS1; *Clu*^*+/−*^ showed a marked increase in astrogliosis assessed by the GFAP immunoreactivity compared with APP/PS1; *Clu*^*+/+*^ littermate controls (Fig. 5a, b and Fig. S7c). We did not observe a significant difference in the number of GFAP-positive astrocytes between APP/PS1; *Clu*^*+/+*^ and APP/PS1; *Clu*^*+/−*^ mice after GFAP immunostaining was normalized to amyloid load (Fig. S7d). Similarly, the evaluation of IBA1 immunoreactivity showed significant differences between CLU genotypes, with CLU haploinsufficient mice having higher levels of microgliosis (Fig. 5d, e and Fig. S7e). No significant differences in the levels of microgliosis between APP/PS1; *Clu*^*+/+*^ and APP/PS1; *Clu*^*+/−*^ animals were observed when normalized to fibrillar amyloid (Fig. S7f). Finally, real-time quantitative PCR revealed an increase in the *Gfap* transcript level in APP/PS1; *Clu*^*+/−*^ animals compared to APP/PS1; *Clu*^*+/+*^ mice (Fig. 5c). Taken together, these data show that partial yet physiological reduction of CLU expression increases severity of amyloid pathology and exacerbates amyloid-associated neurotoxicity and gliosis.

## Discussion

For over three decades the relationship between CLU and Aβ has been studied. Early discoveries of CLU upregulation in AD patients [20], its ability to interact with Aβ [10], and strong genetic association of the *CLU* variants with AD risk [8, 9], have fueled subsequent studies focusing on the mechanism underlying the CLU contribution to AD pathogenesis. Despite the tremendous progress in our understanding of CLU biology, the complexity of its function in the context of AD is still not fully understood. This study was aimed to further elucidate the role of CLU in AD with the focus on physiologic manipulation of expression and the resulting impact on the pathological accumulation of amyloid in the brain.

Initial reports have shown that CLU upregulation significantly correlated with AD [20], prompting the speculation of neuroprotective role of CLU. GWAS studies have linked the protective *T* allele of the common *CLU* rs11136000 SNP with increased CLU levels [8, 9, 22]. In addition, longitudinal analysis of individuals with mild cognitive impairment (MCI) has identified a significant association between increased plasma CLU and slower rates of brain atrophy in multiple brain regions [29, 30], further suggesting protective mechanisms of CLU upregulation in the early stages of the disease development. Moreover, upregulated CLU in plasma correlated with CLU levels in AD vulnerable brain regions [29], suggesting that peripheral CLU mirrors the response of CLU to the pathological changes in the brain. By contrast, the evaluation of CLU in CSF in non-demented elderly individuals showed a strong link of increased CLU with greater atrophy of the entorhinal cortex in CSF Aβ_42_-positive but not in CSF Aβ_42_-negative individuals [31]. Furthermore, the association between increased levels of plasma CLU and atrophy of the entorhinal cortex and hippocampus in AD patients has also been reported [23]. Elevated plasma CLU was also linked to a faster disease progression and more severe AD [21, 23]. Functional studies in mouse models by us [17] and others [15, 16, 18] utilizing whole body, complete knockouts (*Clu*^*−/−*^ mice) have suggested that CLU drives amyloid pathology in brain parenchyma. These conflicting results led us to assess the effect of manipulating CLU levels in a physiologic context on amyloid pathology. We found that viral-induced ~ 30% CLU overexpression in astrocytes resulted in significantly lower amount of amyloid deposited in the form of diffuse and fibrillar plaques in cortex and hippocampus when compared to controls in APP/PS1 mice. In addition, the reduction of amyloid burden was concomitant with decreased neuritic dystrophy surrounding amyloid plaques in APP/PS1^AAV-CLU^ mice compared with their littermate controls. Interestingly, the beneficial effect of increasing peripheral CLU levels has been recently tested in the context of amyloidosis. Subchronic intravenous treatment of 14-month-old APP23 mice with human recombinant CLU led to a significant reduction in insoluble Aβ_40_ and Aβ_42_, with a marked effect on the size of amyloid plaques [32]. In addition, a peripheral increase of CLU was accompanied by lower levels of CAA in the brains of APP23 mice compared to animals treated with saline [32]. Another study has recently evaluated the effect of intracerebroventricular delivery of synthetic CLU peptide in 8-month-old 5XFAD transgenic mice on amyloid pathology and found that that two-week infusion of CLU peptide significantly lowered the amount of amyloid plaques and CAA [33]. We did not detect significant changes in amyloid load in cerebral vessels. One possibility is that an increased secretion of CLU by astrocytes predominantly affects parenchymal Aβ deposits, whereas peripheral delivery of CLU may result in localized effects in blood vessels. In fact, the authors have observed immunoreactivity of human CLU in leptomeningeal vessels [32] which may suggest that CLU presence in the vessels directly stimulates Aβ clearance, leading to a decrease in CAA. This aligns with our previous study demonstrating that complete loss of CLU using *Clu*^*−/−*^ mice promotes the formation of CAA [17].

This study is the first to evaluate the effect of CLU haploinsufficiency on pathological changes in the brain. We have shown that partial loss of CLU by ~ 50% resulted in exaggerated amyloid accumulation in brain parenchyma with significant effects on neurotoxicity and gliosis. This agrees with previous studies showing that CLU increases Aβ solubility and prevents Aβ aggregation. Moreover, it has recently been suggested that the molar ratio of CLU to Aβ is a critical factor in determining Aβ aggregation, with a higher CLU:Aβ ratio promoting Aβ solubilization and lower CLU:Aβ ratio facilitating Aβ accumulation in the brain [34]. Interestingly, recent sequencing studies have found numerous non-synonymous variants and small insertions/deletions within the CLU β-chain region (e.g. p.R338W, p.I360N, p.I303NfsX13, p.T445_D447del) [35]. In vitro studies, that aimed to elucidate the nature of these rare mutations enriched in AD patients, have reported associations between CLU variants and reduced levels of secreted CLU [36].

Taken together, our findings highlight a prominent function of CLU in regulating amyloid aggregation in the brain. In particular, we uncovered neuroprotective properties of CLU overexpression, that results in a significant reduction of amyloid pathology and overall improvement of amyloid-related pathological features, including neurotoxicity and gliosis. Moreover, our study is the first to show that CLU haploinsufficiency leads to more severe amyloidosis. Future investigations should further address how CLU overexpression provides neuroprotection in Aβ-mediated disorders.

## Conclusions

No effective treatments to prevent or slow the progression of AD have been developed to date. However, Aβ has long been considered a promising target for therapeutic interventions. Our study provides a strong evidence that ~ 30% CLU overexpression ameliorates amyloid pathology while ~ 50% reduction of CLU exacerbates amyloid accumulation in the brain, thus reflecting a protective role of CLU in AD. Given that increasing CLU levels has potential consequences for Aβ-related therapies, future studies determining the exact mechanism and factors modulating CLU expression are critical. Our findings also indicate that pharmacological or gene delivery approaches aimed at increasing the levels of CLU in the brain could be a viable therapeutic strategy for combating AD.

## Supplementary Information

**Additional file 1: Figure S1.** Widespread expression of AAV-GFP in APP/PS1 mice. **a** GFP expression observed in cortex and hippocampus followed by viral transduction of AAV-GFP at postnatal day 2.

**Additional file 2: Figure S2.** CLU overexpression in astrocytes is associated with reduced levels of total amyloid in APP/PS1 mice. **a** Representative images showing the Aβ immunoreactivity in cortex and hippocampus of APP/PS1 mice. Scale bar, 100 μm.

**Additional file 3: Figure S3.** Increased CLU levels do not influence amyloid deposition in cerebrovasculature. **a** Amyloid deposits were quantified in leptomeningeal vessels. **b** Stereological quantification of amyloid aggregation in cortical and hippocampal arteries and arterioles. *N*=11-12 mice/group. For each animal three brain sections were analyzed. Data are presented as mean ± S.E.M. and analyzed by Student’s *t* test.

**Additional file 4: Figure S4.** CLU upregulation impacts amyloid-associated neurotoxicity and inflammation. **a** Representative images of neuritic dystrophy surrounding amyloid plaques in cortex of APP/PS1 mice. Scale bar, 50 μm. **b** The levels of Lamp1 were normalized to fibrillar amyloid. *N* = 10 mice/group. Data are presented as mean ± S.E.M. and Student’s *t* tests were used to analyze cortex and hippocampus. **c** Astrogliosis is observed in close proximity to fibrillar plaques. Scale bar, 50 μm. **d** GFAP staining was normalized to the amount of fibrillar amyloid. *N* = 10 mice/group. Data are presented as mean ± S.E.M. and analyzed by Student’s *t* test. **e** IBA1 staining was used to mark microgliosis. Scale bar, 50 μm. **f** IBA1 levels were normalized to fibrillar amyloid plaques. *N* = 10 mice/group. Data are presented as mean ± S.E.M. and analyzed by Student’s *t* test.

**Additional file 5: Figure S5.** Augmented deposition of total amyloid in APP/PS1; *Clu*^*+/−*^ mice. **a** Representative images of total Aβ deposition in cortex and hippocampus of APP/PS1 mice. Scale bar, 100 μm.

**Additional file 6: Figure S6.** CLU reduction does not increase CAA. **a** Stereological analysis of the CAA level in leptomeningeal vessels. **b** Quantification of amyloid load in penetrating blood vessels in APP/PS1 animals. *N* = 7–8 mice/group. For each animal three brain sections were analyzed. Data are presented as mean ± S.E.M. and analyzed by Student’s *t* tests.

**Additional file 7: Figure S7.** CLU haploinsufficiency increases the levels of neuritic dystrophy and gliosis. **a** Amyloid-associated neuritic dystrophy surrounding amyloid plaques in cortex of APP/PS1; *Clu*^*+/+*^ and APP/PS1; *Clu*^*+/−*^ mice. Scale bar, 50 μm. **b** The levels of Lamp1 were normalized to fibrillar amyloid. *N* = 7–8 mice/group. Data are presented as mean ± S.E.M. and Student’s *t* tests were used to analyze cortex and hippocampus. **c** More abundant astrogliosis is observed in APP/PS1; *Clu*^*+/−*^ mice compared to controls. Scale bar, 50 μm. **d** GFAP levels were normalized to fibrillar plaques. *N* = 7–8 mice/group. Data are presented as mean ± S.E.M. and analyzed by Student’s *t* test. **e** Microgliosis surrounds amyloid plaques in brain parenchyma. Scale bar, 50 μm. **f** IBA1 immunoreactivity was normalized to fibrillar amyloid. *N* = 7–8 mice/group. Data are presented as mean ± S.E.M. and analyzed by Student’s *t* test.

## Data Availability

All data used and analyzed for the current study are available from the corresponding author on reasonable request.

## Abbreviations

AAV: Adeno-associated viral vectors
Aβ: Amyloid- β peptide
AD: Alzheimer’s disease
CLU: Clusterin
GFAP: Glial fibrillary acidic protein
IBA1: Ionized calcium binding adaptor molecule 1
Lamp1: Lysosomal-associated membrane protein 1
NeuN: Neuronal nuclear protein
SNP: Single nucleotide polymorphism
TBS: Tris-buffered saline
