# Supplementary figures and images for: Astrocyte-derived clusterin suppresses amyloid formation in vivo
Source: Mol Neurodegener. 2020 Nov 27;15:71. doi: 10.1186/s13024-020-00416-1 (PMC7694353; doi:10.1186/s13024-020-00416-1)

**a**

APP/PS1<sup>AAV-GFP</sup>

Cortex

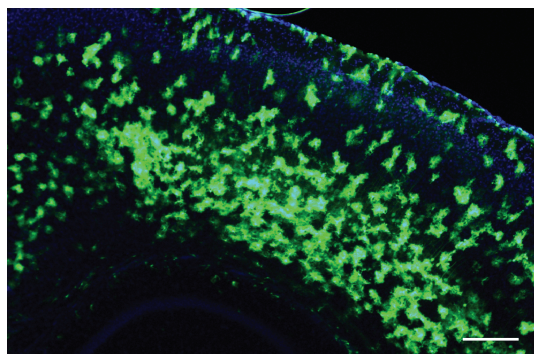

Hippocampus

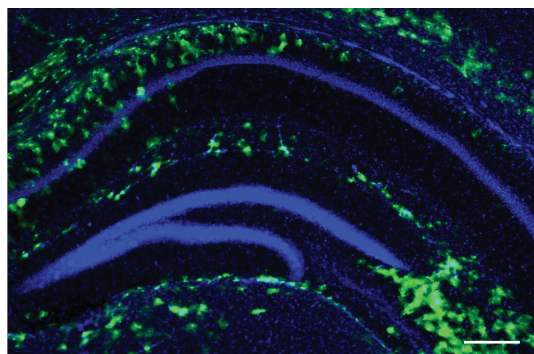

Supplement: Supplementary file 1 — Additional file 1: Figure S1. Widespread expression of AAV-GFP in APP/PS1 mice. a GFP expression observed in cortex and hippocampus followed by viral transduction of AAV-GFP at postnatal day 2. [file 13024_2020_416_MOESM1_ESM.pdf]

APP/PS1<sup>AAV-GFP</sup>

APP/PS1<sup>AAV-CLU</sup>

Cortex

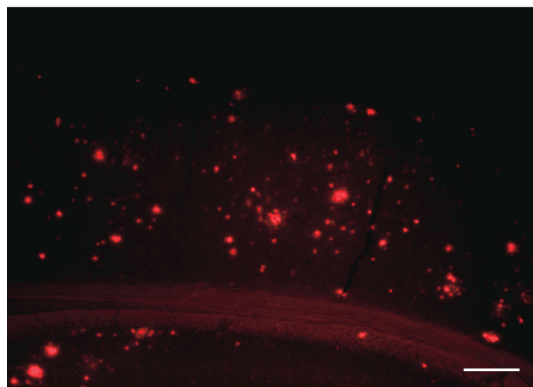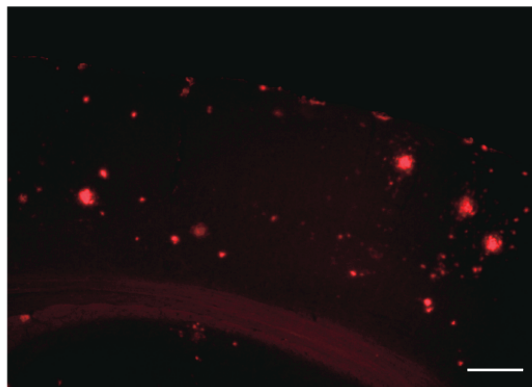

Hippocampus

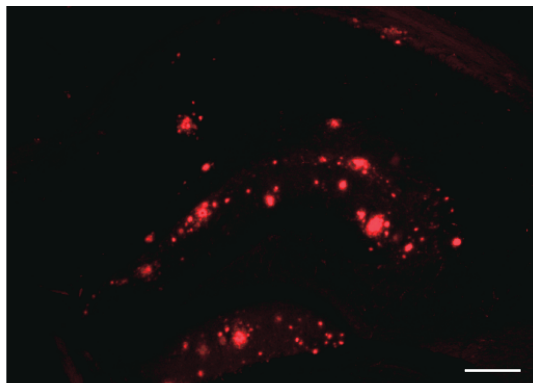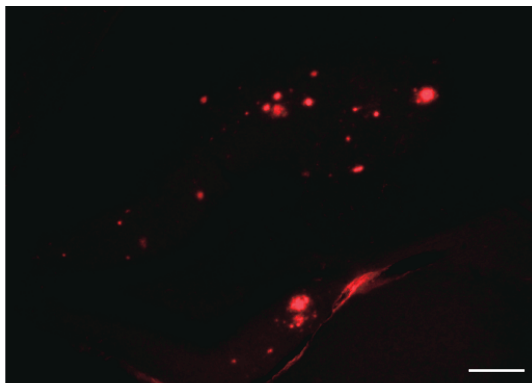

Supplement: Supplementary file 2 — Additional file 2: Figure S2. CLU overexpression in astrocytes is associated with reduced levels of total amyloid in APP/PS1 mice. a Representative images showing the Aβ immunoreactivity in cortex and hippocampus of APP/PS1 mice. Scale bar, 100 μm. [file 13024_2020_416_MOESM2_ESM.pdf]

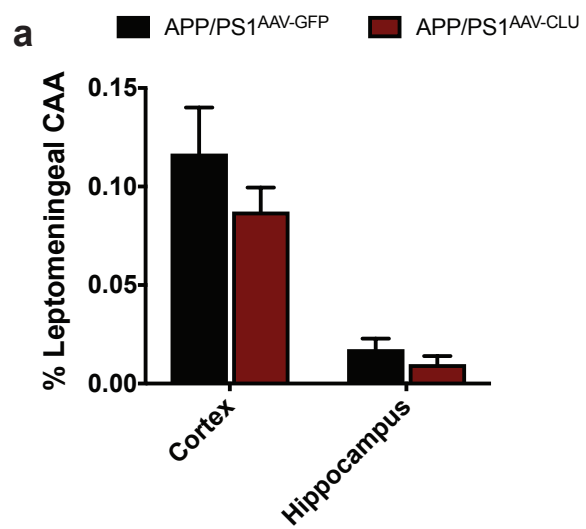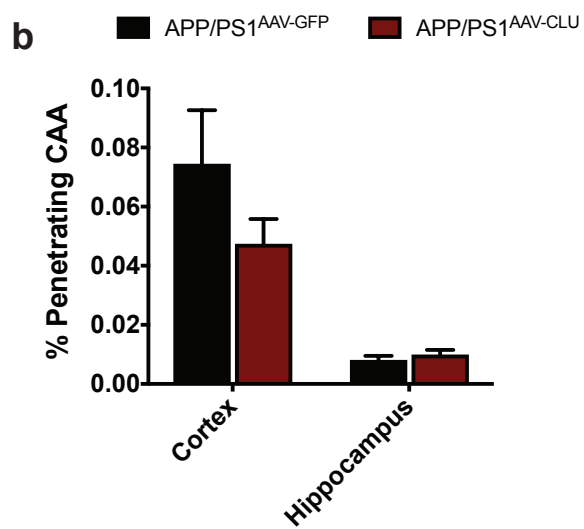

Supplement: Supplementary file 3 — Additional file 3: Figure S3. Increased CLU levels do not influence amyloid deposition in cerebrovasculature. a Amyloid deposits were quantified in leptomeningeal vessels. b Stereological quantification of amyloid aggregation in cortical and hippocampal arteries and arterioles. N=11-12 mice/group. For each animal three brain sections were analyzed. Data are presented as mean ± S.E.M. and analyzed by Student’s t test. [file 13024_2020_416_MOESM3_ESM.pdf]

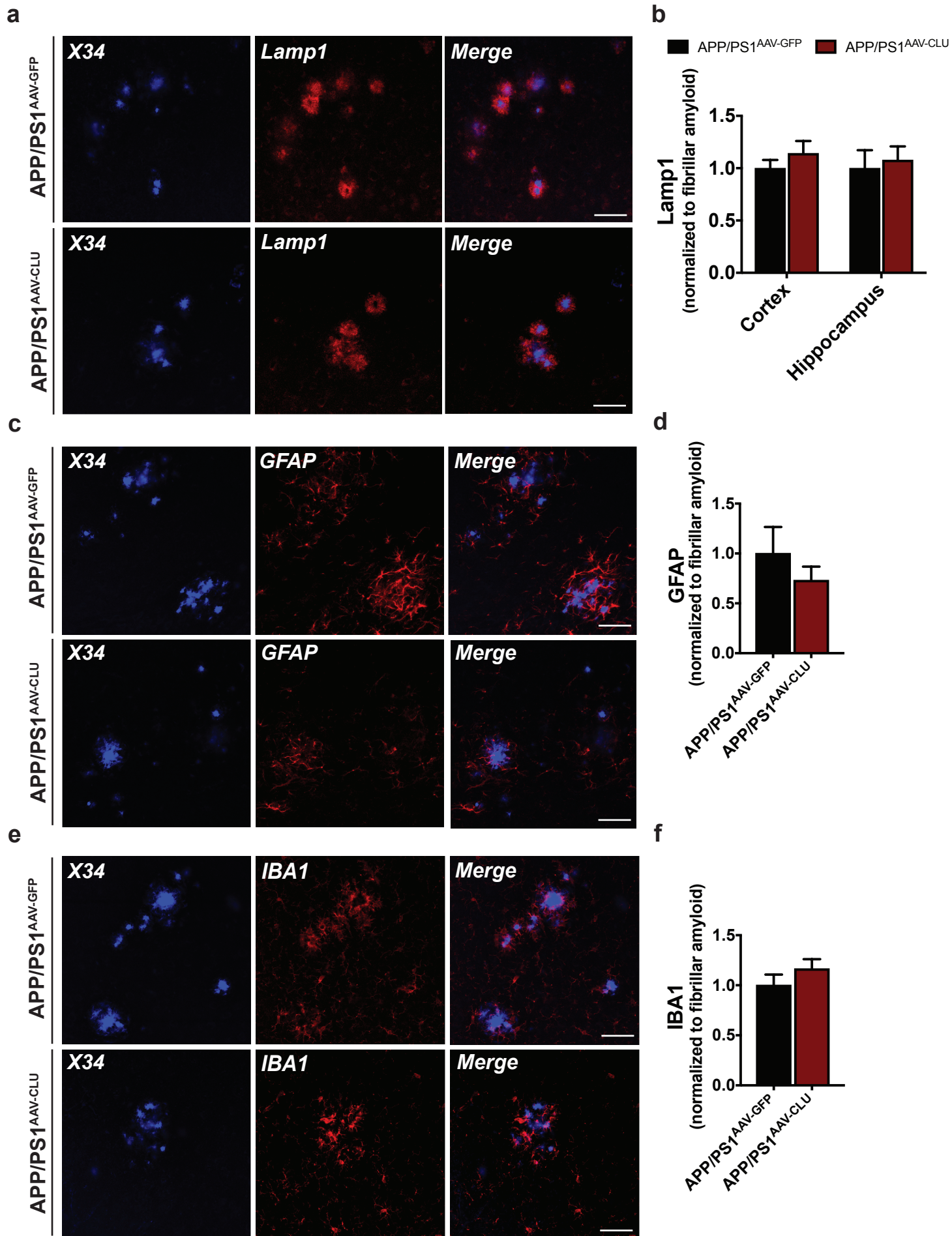

Supplement: Supplementary file 4 — Additional file 4: Figure S4. CLU upregulation impacts amyloid-associated neurotoxicity and inflammation. a Representative images of neuritic dystrophy surrounding amyloid plaques in cortex of APP/PS1 mice. Scale bar, 50 μm. b The levels of Lamp1 were normalized to fibrillar amyloid. N = 10 mice/group. Data are presented as mean ± S.E.M. and Student’s t tests were used to analyze cortex and hippocampus. c Astrogliosis is observed in close proximity to fibrillar plaques. Scale bar, 50 μm. d GFAP staining was normalized to the amount of fibrillar amyloid. N = 10 mice/group. Data are presented as mean ± S.E.M. and analyzed by Student’s t test. e IBA1 staining was used to mark microgliosis. Scale bar, 50 μm. f IBA1 levels were normalized to fibrillar amyloid plaques. N = 10 mice/group. Data are presented as mean ± S.E.M. and analyzed by Student’s t test. [file 13024_2020_416_MOESM4_ESM.pdf]

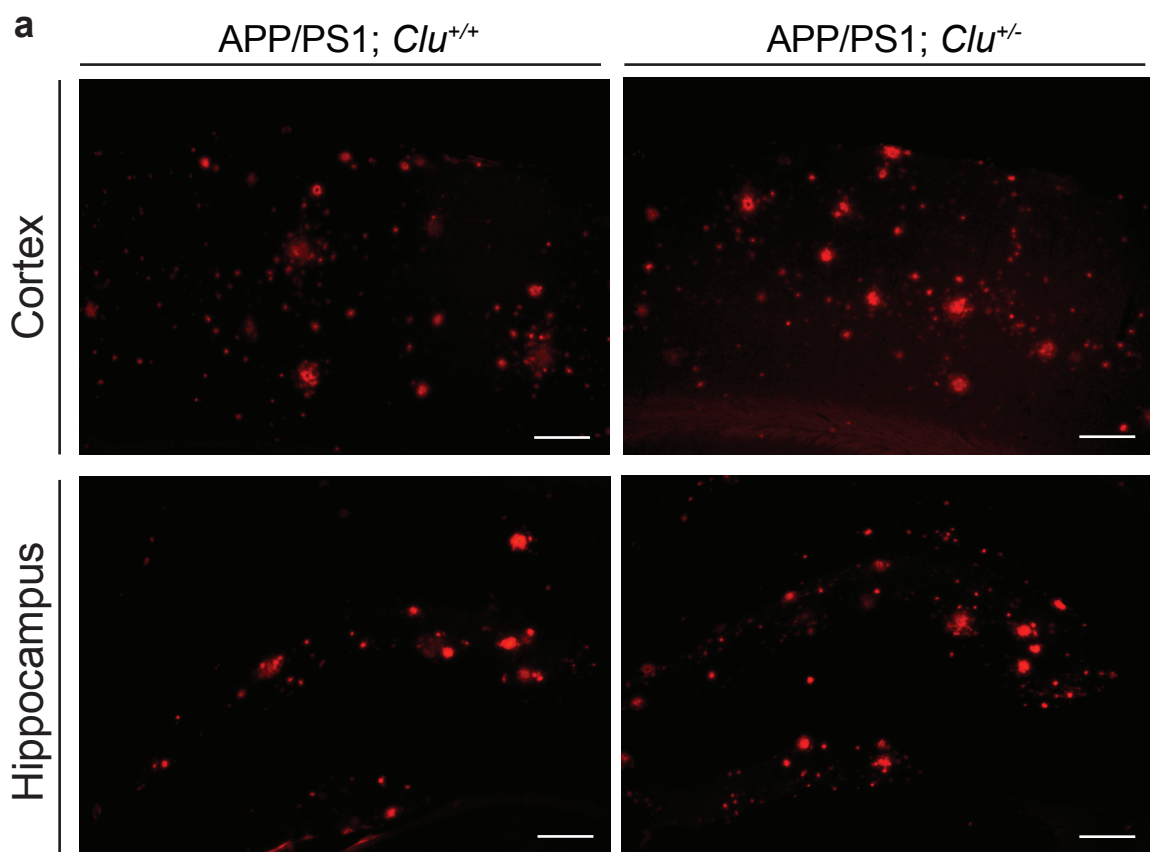

Supplement: Supplementary file 5 — Additional file 5: Figure S5. Augmented deposition of total amyloid in APP/PS1; Clu+/− mice. a Representative images of total Aβ deposition in cortex and hippocampus of APP/PS1 mice. Scale bar, 100 μm. [file 13024_2020_416_MOESM5_ESM.pdf]

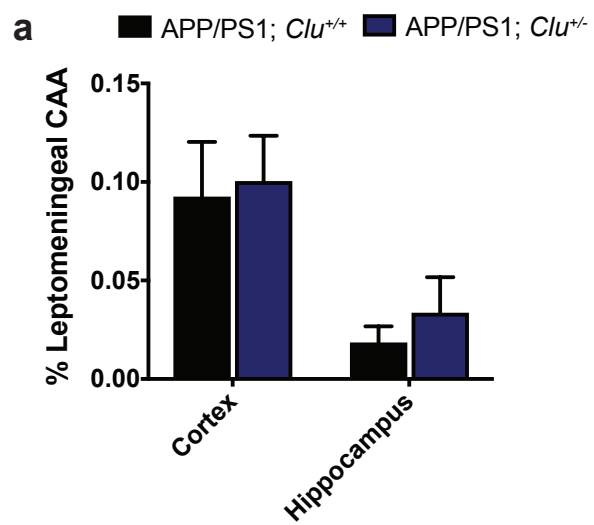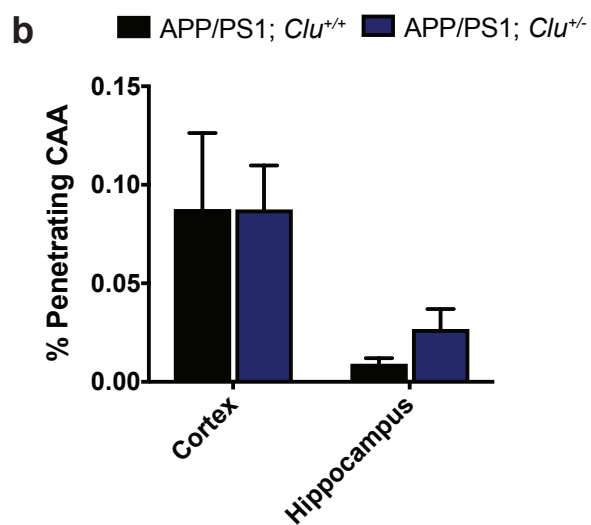

Supplement: Supplementary file 6 — Additional file 6: Figure S6. CLU reduction does not increase CAA. a Stereological analysis of the CAA level in leptomeningeal vessels. b Quantification of amyloid load in penetrating blood vessels in APP/PS1 animals. N = 7–8 mice/group. For each animal three brain sections were analyzed. Data are presented as mean ± S.E.M. and analyzed by Student’s t tests. [file 13024_2020_416_MOESM6_ESM.pdf]

**a**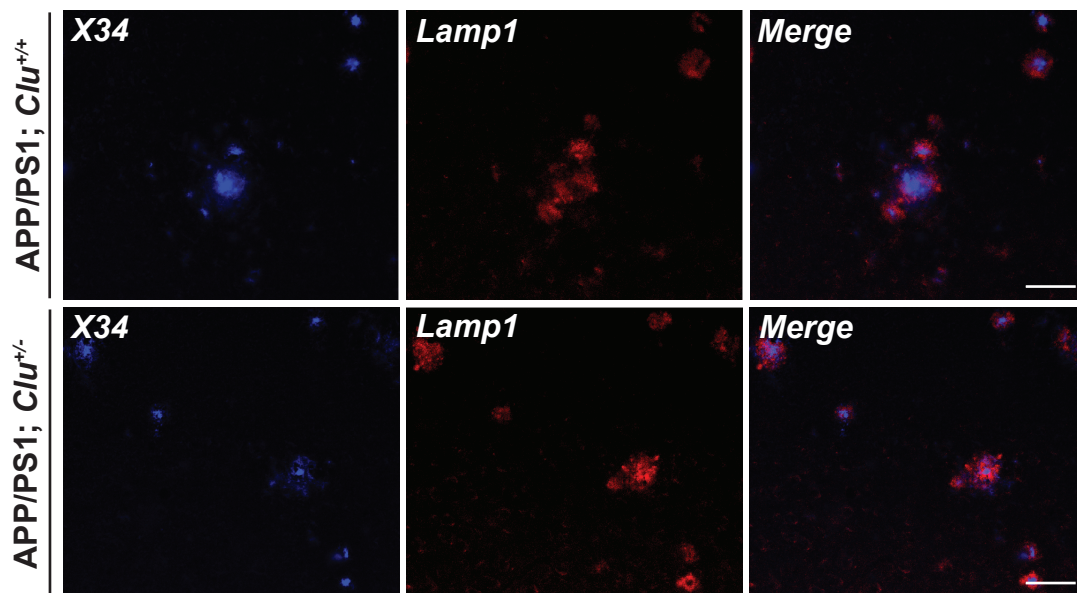**b**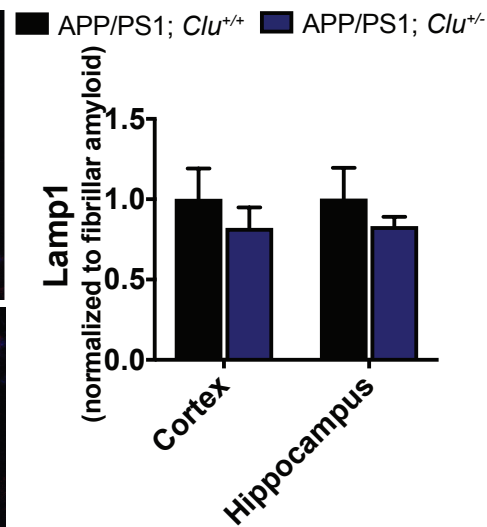**c**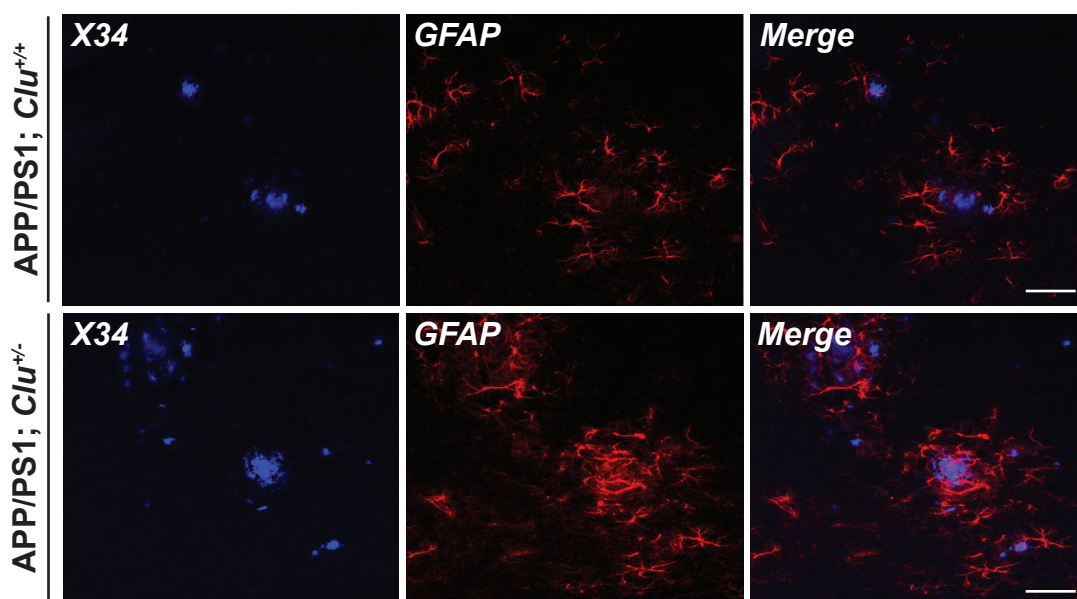**d**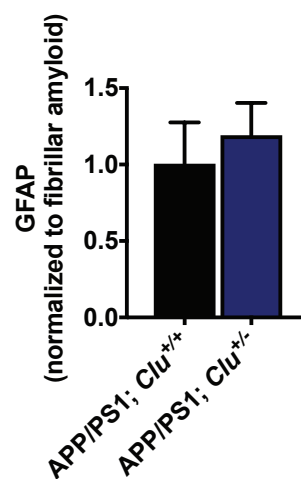**e**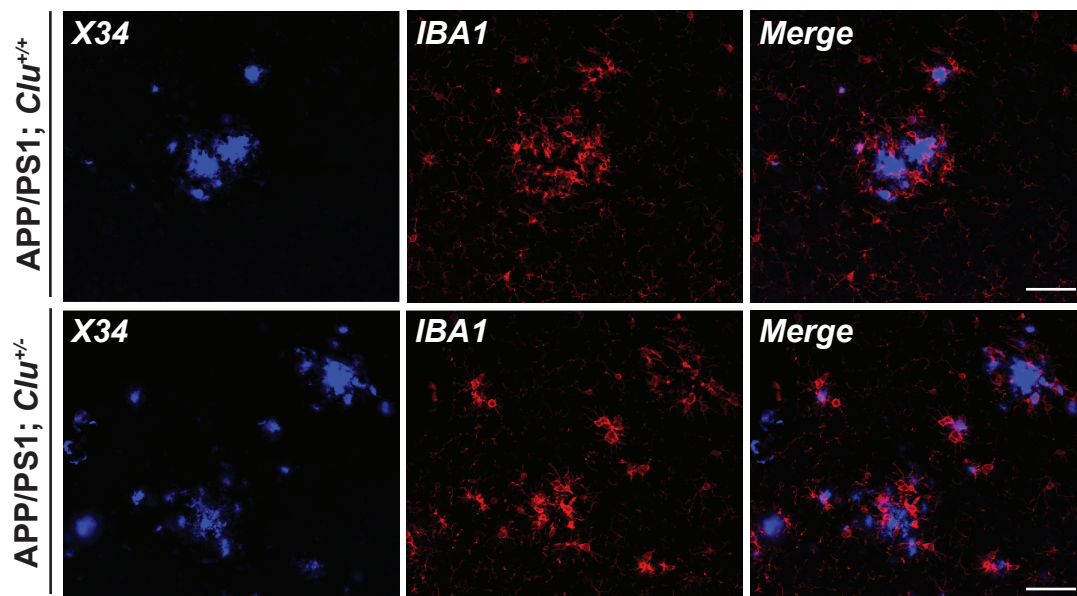**f**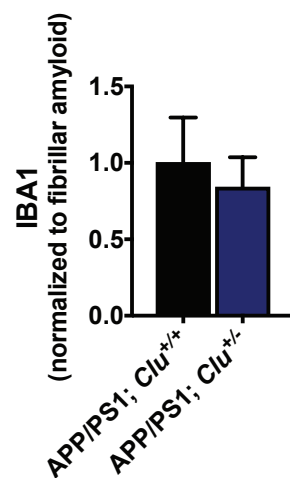

Supplement: Supplementary file 7 — Additional file 7: Figure S7. CLU haploinsufficiency increases the levels of neuritic dystrophy and gliosis. a Amyloid-associated neuritic dystrophy surrounding amyloid plaques in cortex of APP/PS1; Clu+/+ and APP/PS1; Clu+/− mice. Scale bar, 50 μm. b The levels of Lamp1 were normalized to fibrillar amyloid. N = 7–8 mice/group. Data are presented as mean ± S.E.M. and Student’s t tests were used to analyze cortex and hippocampus. c More abundant astrogliosis is observed in APP/PS1; Clu+/− mice compared to controls. Scale bar, 50 μm. d GFAP levels were normalized to fibrillar plaques. N = 7–8 mice/group. Data are presented as mean ± S.E.M. and analyzed by Student’s t test. e Microgliosis surrounds amyloid plaques in brain parenchyma. Scale bar, 50 μm. f IBA1 immunoreactivity was normalized to fibrillar amyloid. N = 7–8 mice/group. Data are presented as mean ± S.E.M. and analyzed by Student’s t test. [file 13024_2020_416_MOESM7_ESM.pdf]
